# Structural and functional alterations of the tracheobronchial tree after left upper pulmonary lobectomy for lung cancer

**DOI:** 10.1186/s12938-019-0722-6

**Published:** 2019-10-25

**Authors:** Qingtao Gu, Shouliang Qi, Yong Yue, Jing Shen, Baihua Zhang, Wei Sun, Wei Qian, Mohammad Saidul Islam, Suvash C. Saha, Jianlin Wu

**Affiliations:** 1 Sino-Dutch Biomedical and Information Engineering School, Northeastern University, Shenyang, China; 2 Key Laboratory of Medical Image Computing of Northeastern University (Ministry of Education), Shenyang, China; 3 Department of Radiology, Shengjing Hospital of China Medical University, Shenyang, China; 4 Department of Radiology, Affiliated Zhongshan Hospital of Dalian University, Dalian, China; 5 The Graduate School, Dalian Medical University, Dalian, China; 6 College of Engineering, University of Texas at El Paso, El Paso, USA; 7 School of Mechanical and Mechatronic Engineering, Faculty of Engineering and Information Technology, University of Technology Sydney, Brisbane, Australia

**Keywords:** Pulmonary lobectomy, Lung cancer, Tracheobronchial tree, CT, Computational fluid dynamics, Bronchial distortion, Pressure drop

## Abstract

**Background:**

Pulmonary lobectomy has been a well-established curative treatment method for localized lung cancer. After left upper pulmonary lobectomy, the upward displacement of remaining lower lobe causes the distortion or kink of bronchus, which is associated with intractable cough and breathless. However, the quantitative study on structural and functional alterations of the tracheobronchial tree after lobectomy has not been reported. We sought to investigate these alterations using CT imaging analysis and computational fluid dynamics (CFD) method.

**Methods:**

Both preoperative and postoperative CT images of 18 patients who underwent left upper pulmonary lobectomy are collected. After the tracheobronchial tree models are extracted, the angles between trachea and bronchi, the surface area and volume of the tree, and the cross-sectional area of left lower lobar bronchus are investigated. CFD method is further used to describe the airflow characteristics by the wall pressure, airflow velocity, lobar flow rate, etc.

**Results:**

It is found that the angle between the trachea and the right main bronchus increases after operation, but the angle with the left main bronchus decreases. No significant alteration is observed for the surface area or volume of the tree between pre-operation and post-operation. After left upper pulmonary lobectomy, the cross-sectional area of left lower lobar bronchus is reduced for most of the patients (15/18) by 15–75%, especially for 4 patients by more than 50%. The wall pressure, airflow velocity and pressure drop significantly increase after the operation. The flow rate to the right lung increases significantly by 2–30% (but there is no significant difference between each lobe), and the flow rate to the left lung drops accordingly. Many vortices are found in various places with severe distortions.

**Conclusions:**

The favorable and unfavorable adaptive alterations of tracheobronchial tree will occur after left upper pulmonary lobectomy, and these alterations can be clarified through CT imaging and CFD analysis. The severe distortions at left lower lobar bronchus might exacerbate postoperative shortness of breath.

## Background

Lung cancer has been the most common cancer worldwide in terms of both incidence and mortality. In 2012, there were 1.82 million new cases accounting for about 13.0% of the total number of new cases, and 1.56 million deaths representing 19.4% of all deaths from cancer [1]. Pulmonary lobectomy, especially Video-assisted thoracoscopic surgery (VATS) lobectomy, is a well-established curative treatment method for localized lung cancer [2, 3].

Pulmonary lobectomy results in a permanent loss of pulmonary function. Normally, this loss is proportional to the volume of resected lung, but it is also affected by the adaptive remodeling of the remaining lung. In the upper lobectomy, the upward displacement of the diaphragm and the remaining lobe will make the ipsilateral bronchus distort anatomically in a sigmoidal form, thereby resulting in the bronchial angulation. If the resultant stenosis is higher than 80%, a bronchial kink occurs [4]. The stenosis will result in lower postoperative functional lung volume (FLV) and postoperative forced expiratory volume in 1 s (FEV_1_), which will lead to some complications characterized by the shortness of breath and persistent cough.

The high-resolution computed tomography (CT) images are used for the anatomic alterations and postoperative complications [5–7]. Ueda et al. initially reported that bronchial kink was found in 42% (21/50) of the patients and bronchial kink may exacerbate the postoperative deterioration of lung function [4]. It has been proved that CT-based bronchography can help to screen the bronchial kink without additional invasive study. Seok et al. found that the increased angle of the bronchi is associated with the decline of pulmonary function [8]. Sengul et al. demonstrated that the changes of postoperative lung volume depend on the resected lobe [9]. Specifically, for the lower lobectomy, the reduction of the total lung volume is less than that of the upper lobectomy. However, the general pattern of structural alterations of the tracheobronchial tree, specifically for the left upper pulmonary lobectomy (estimated to account for one-third of all cancer [10]) has not been reported.

The changes of postoperative pulmonary functions are measured by the spirometry-based pulmonary function tests (PFTs) [11]. The expansion of both the contralateral lung and the remaining ipsilateral lung contributes to the postoperative compensation of pulmonary function [9]. This kind of compensation depends on the resected lobe and is more robust after lower lobectomy [12]. However, the postoperative pulmonary function can be underestimated by only the measure of FEV_1_ through PFTs [13]. Moreover, postoperative PFT is not routinely performed for all patients, it needs the cooperation of the patients and it is not suitable for the patients with breathlessness. For example, only 60 among 202 patients who underwent lobectomy had PFT in the study by Ueda et al. [12].

Depending on the individualized structural models of the tracheobronchial tree extracted from CT images, the computational fluid dynamics (CFD) simulation can provide physiologically significant ventilation information including the airflow velocity, wall pressure, wall shear stress, pressure drop and lobular airflow rate, which may complement the results of anatomy and pulmonary function [14–17]. Walters et al. proposed to use the reduced geometry model to reduce the complexity [18]. Oakes et al. investigated the effect of age on the airflow pattern and airway resistance [19] and Sul et al. assessed the airflow sensitivity on the lobar flow fraction [20]. Turbulent characteristics have been observed downstream of the glottis by Calmet et al. [21]. It has been reported that the obstructions in the lower airway caused bronchial tumor or other lesion can alter airflow patterns in the central airway [22, 23]. In our previous work, CFD simulations have been done to study airflow characteristics in subjects with left pulmonary artery sling, the tracheal bronchus and chronic obstructive pulmonary disease [24–28]. Besides the studies on the flow in the airway tree models with asthma and severe stenosis, CFD has also been used to facilitate various treatments, such as acute bronchodilation in asthmatics, tracheobronchial stent placement, vascular ring surgery and antibiotic treatment with cystic fibrosis [29–32]. It should be noted that the results of CFD simulation have been validated by both in vitro experiments and in vivo SPECT/CT images [33, 34].

The contributions of this work are summarized as follows. First, the structural alterations of the tracheobronchial trees after left upper pulmonary lobectomy for lung cancer are investigated through various quantitative measures including the angles between trachea and bronchi, the surface area and volume of the tree, and the cross-sectional area of the left lower lobar bronchus. Second, the alterations of the airflow are characterized by CFD-based measures of the wall pressure, airflow velocity, pressure drop, lobar flow rate, and local flow features at the left lower lobar bronchus. Third, the relationship between alterations of airway structure and ventilation function is illustrated. To the best of our knowledge, this is the first systematic study which combines quantitative CT images and CFD analysis to clarify the structural and functional alterations of the tracheobronchial tree caused by left upper pulmonary lobectomy.

## Results

### Structural alterations of the tracheobronchial tree

**Fig. 1:**
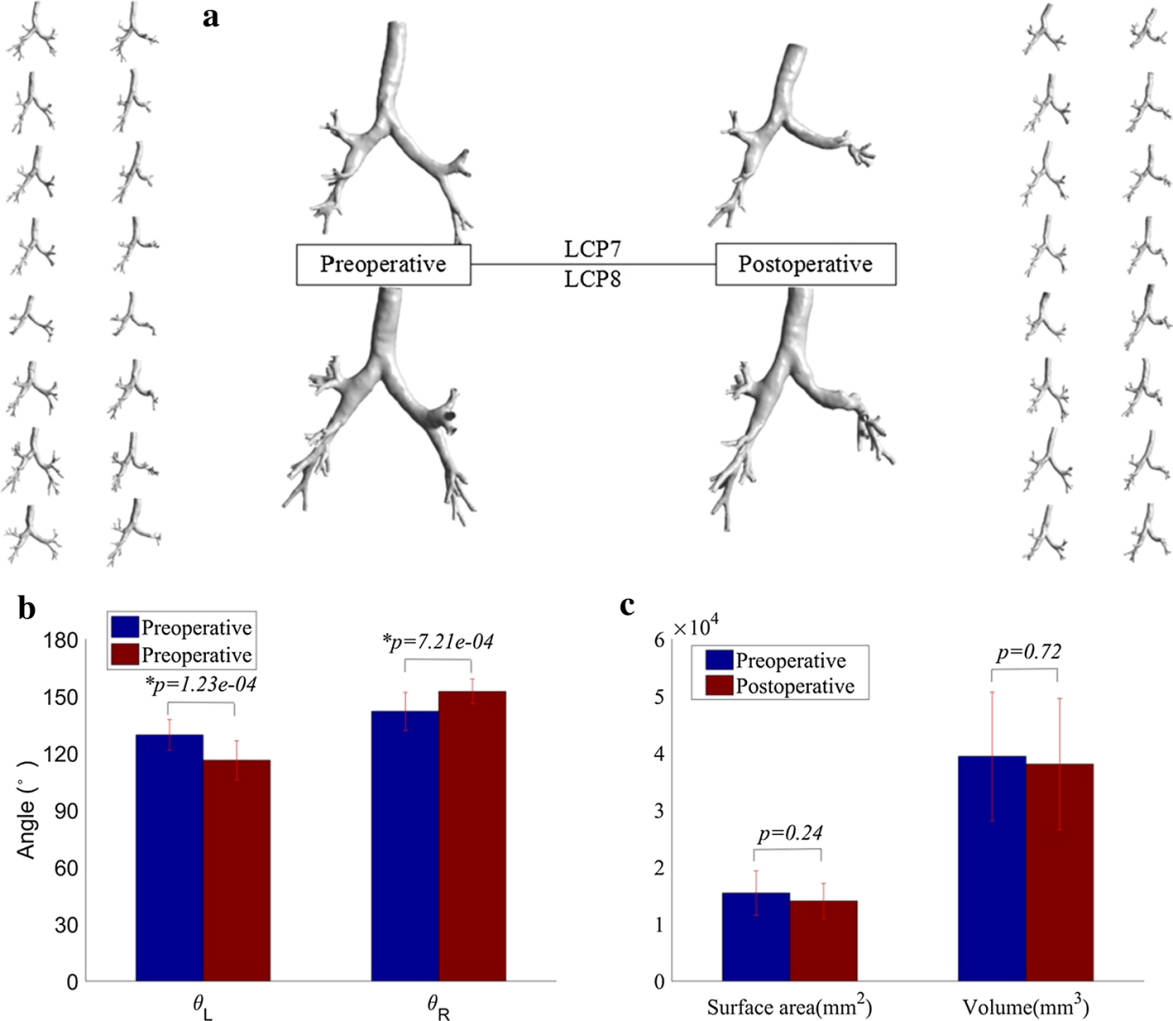
Structural alterations of tracheobronchial trees after the left upper pulmonary lobectomy. **a** The global appearances. **b** The angles between the trachea and the main bronchus. **c** The volume and surface area

Postoperatively, the global alterations (deformation) of the tracheobronchial tree can be found in Fig. 1a. It is in agreement with previous observation that the left main bronchus distorts in a sigmoidal form [4], as a result of the upward displacement of diaphragm and the remaining left lower lobe. In addition, the trachea seems to slant to the left and the stenosis occurs at the left lower lobar bronchus, but not at the left main bronchus. However, neither for the volume nor the surface area, there is no significant difference between preoperative and postoperative tracheobronchial trees, as shown in Fig. 1c.

Compared with the preoperative models, the angle between the trachea and the left main bronchus ($\theta_{\text{L}}$) decreases significantly in the postoperative models (*p *< 0.01), by the mean of 13.4°. Nonetheless, $\theta_{\text{R}}$ increases significantly by the mean of 10.5 degrees as shown in Fig. 1b. These alterations are thought to be associated with the upward displacement of diaphragm and the remaining lobe.

**Fig. 2:**
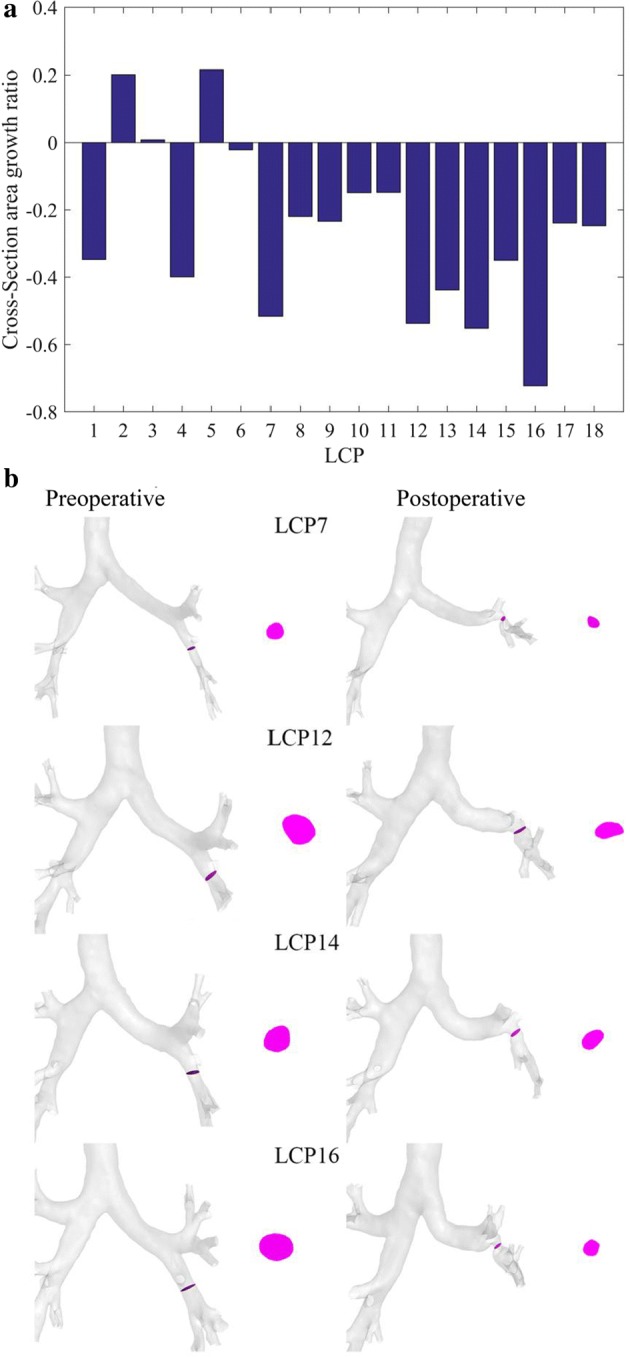
Alterations of the left lower lobar bronchus induced by the left upper pulmonary lobectomy. **a** The cross-sectional area growth rate for all patients. **b** The alterations of the left lower lobar bronchus

The cross-sectional area growth rate ($R$) is given for each patient in Fig. 2a. It is found that $R$ is negative for most patients (15/18), indicating that the left lower lobar bronchus becomes narrow (15–75%) after lobectomy. For four patients (LCP7, LCP12, LCP14 and LCP16), the stenosis is higher than 50%. The location and cross section of the stenosis are given in Fig. 2b.

### Alterations of airflow in the tracheobronchial tree

#### Wall pressure and flow velocity distribution

**Fig. 3:**
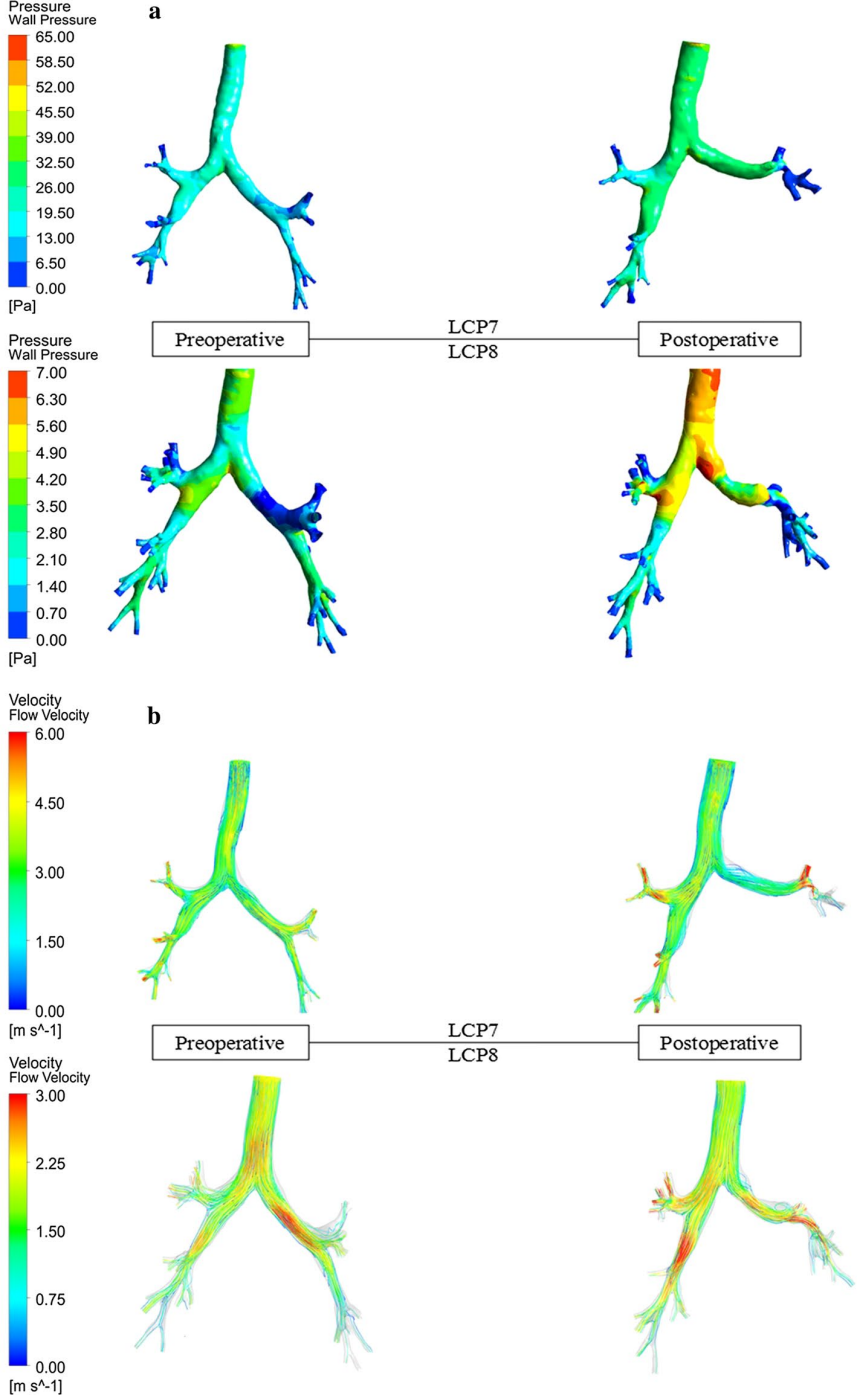
The wall pressure and flow velocity in preoperative and postoperative tracheobronchial trees. **a** LCP7 with a 51.64% stenosis at the left lower lobar bronchus. **b** LCP8 with a 21.95% stenosis at the left lower lobar bronchus

The wall pressure distribution is given in Fig. 3a for LCP7 and LCP8 as examples. It can be seen that the wall pressure at the trachea and the main bronchi increases significantly after the lobectomy. The maximum wall pressure in LCP7 reaches 65.0 Pa for the stenosis higher than 50% at the left lower lobar bronchus. For LCP8 with a stenosis of 21.95%, the maximum wall pressure is only about 7.0 Pa. After the left upper lobectomy, the average wall pressure in 17 patients is higher than that before the surgery, with an increase ranging from 0.1747 to 5.7243 Pa. One patient (LCP15) had a decrease of 0.7506 Pa.

Figure 3b presents the flow velocity within the tracheobronchial trees for LCP7 and LCP 8 as examples. The air flow velocity in the left lower lobe increases significantly after lobectomy. Preoperatively, the velocity at the left lower bronchus of LCP7 and LCP8 is 3.00 m/s and 1.50 m/s, respectively; the velocity in postoperative model increases to 4.50 m/s and 2.25 m/s, respectively. The maximum velocity in LCP7 (6.00 m/s) is higher than that in LCP8 (3.00 m/s) due to higher stenosis. After the lobectomy, the maximum airflow velocity within the tracheobronchial tree increases significantly by 0.09–4.26 m/s in 16 patients. For the remaining patients, it has a slight decrease of about 0.76 m/s.

#### Pressure drop

**Fig. 4:**
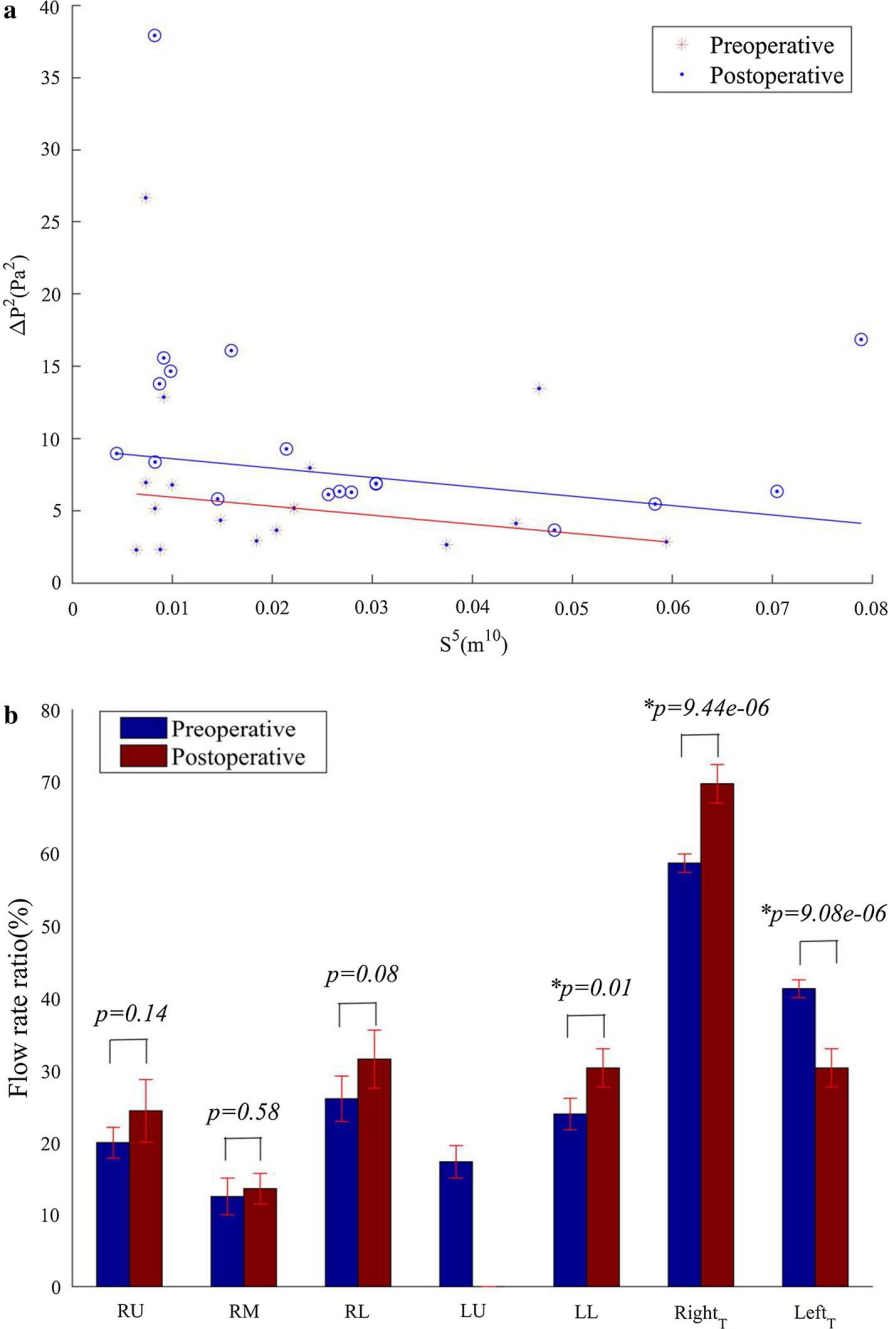
Alterations of pressure drop and airflow rate distribution induced by the left upper pulmonary lobectomy. **a** The pressure drop vs the sectional area of inlet. **b** The airflow rate distribution

The pressure drop can be calculated as the difference between the mean pressure at the inlet of the trachea and the average pressure of the outlet (the atmospheric pressure). According to Eq. (8), the relationship between the pressure drop and the inlet area can be presented in Fig. 4a. After the left upper lobectomy, the pressure drop ($\Delta P$) increased in 16 patients with a range of 0.81–10.37 Pa. In the remaining two patients, $\Delta P$ decreased by 3.90 and 1.62 Pa, respectively. The slopes of the fitting line before and after the lobectomy are roughly the same, indicating that the relationship between the pressure drop and the inlet area remains unchanged. Meanwhile, the postoperative fitting line is above the preoperative one.

#### Airflow rate distribution

The airflow rate for each lobe and left and right lung is given in Fig. 4b. Though the flow rate to the right upper lobe, right middle lobe, and right lobe increases after the lobectomy, no significant difference is available (*p* > 0.01). The postoperative flow rate to the left lower lobe is significantly higher than that before lobectomy (*p* < 0.01) by 6.36% (0.6211 × 10^−4^ kg/s). The postoperative flow rate to the right lung is significantly higher than that before lobectomy (*p* < 0.01) by 10.97%. Preoperatively, the ratio of the airflow rate to the right lung to that to the left lung is 58.67%/41.32%. It turns into 69.65%/30.35% postoperatively.

#### Local alterations

**Fig. 5:**
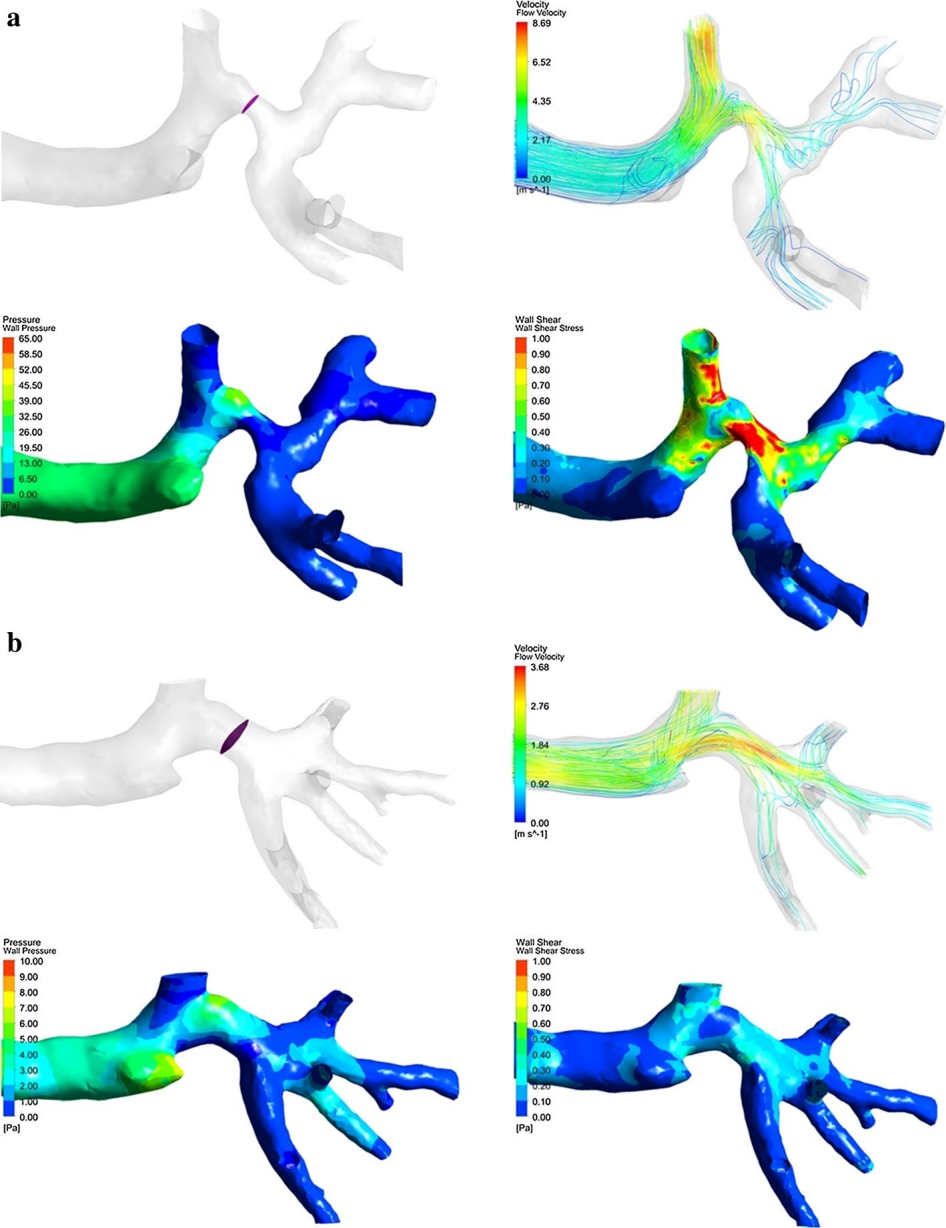
Local structure of the tracheobronchial tree, flow velocity, wall pressure and wall shear stress after the left upper pulmonary lobectomy. **a** LCP7 with a 51.64% stenosis at the left lower lobar bronchus. **b** LCP8 with a 21.95% stenosis at the left lower lobar bronchus

Local alterations of the structure, velocity, wall pressure, and wall shear stress are given in Fig. 5 for LCP7 and LCP8 as examples. For LCP7, there is an increase in the flow velocity at the stenosis of the left lower lobar bronchus and the occurrence of turbulence. A clear vortex appears in the remnants of the left lower lobe, and the streamline is distorted. The wall pressure and wall shear stress increase at the stenosis after lobectomy. For LCP8, the lower stenosis corresponds to the relatively smooth streamlines, small increase of wall pressure and wall shear stress.

## Discussions

The present study characterized the structural and functional alterations of the tracheobronchial tree after left upper pulmonary lobectomy for lung cancer using the preoperative and postoperative CT images of 18 patients. These alterations firstly and comprehensively describe the adaptive remodeling of the remaining respiratory system after the left upper lobectomy. The favorable remodeling includes the increased angle between the trachea and right main bronchus and the significant growth of flow rate ratio to the right lung. The unfavorable remodeling are the decrease of the angle between the trachea and left main bronchus, the sigmoidal distortion of the left main bronchus, and the decrease of sectional area (narrowing) of the left lower lobar bronchus. The narrowing of bronchus, the severe stenosis in particular, increases the flow velocity, the wall pressure, the wall shear stress, the possibility of vortex and the pressure drop; while the inlet boundary condition is the steady constant flow rate for our present simulation. The favorable and unfavorable remodelings lay a foundation for understanding the “compensatory lung adaption” and etiology of postoperative breathless, persistent cough and inflammation. The main findings, the methodological advantages and their significance will be presented as follows.

The first main finding of this study is about the favorable adaptive remodeling of the remaining respiratory system after the left upper lobectomy. $\theta_{\text{R}}$ increases significantly from 142° to 152° and the flow rate ratio increases from 58.67 to 69.65%. The increase of $\theta_{\text{R}}$ facilitates the ventilation of the right lung, resulting in the increase of the flow rate ratio. It partially contributes to “compensatory lung adaption”, one phenomenon that postoperative pulmonary function is better than the estimated one [4]. Sengul et al. reported that after the left upper lobectomy, the ipsilateral and contralateral lung volumes decrease by 39.31% and 2.72%, respectively [9]. For the lower lobectomy, postoperative compensation is obtained by the expansion of both contralateral lung and remaining ipsilateral lung. It is noted that the statistical power of the study by Sengul et al. [9] is low for only five patients with left upper lobectomy are included.

The second main finding of this study is about the unfavorable alterations induced by lobectomy. These alterations include the decrease of the angle between the trachea and left main bronchus, the sigmoidal distortion of the left main bronchus, and the stenosis of the left lower lobar bronchus (the degree of stenosis is greater than 50% in some cases). Despite of these unfavorable alterations, the increased flow rate ratio to the remaining left lower lobe (from 23.98 to 30.34%) demonstrates that the pulmonary function of the left lower lobe is augmented, contributing to the “compensatory lung adaption”. The observations of this study have two aspects different with previous study. Firstly, the bronchial kink (80% stenosis) is not found for the present model. However, according to Ueda et al. [4], the bronchial kink was observed in up to 42% of the patients who had undergone the upper lobectomy. Secondly, the stenosis is not at the left main bronchus, but at the left lower lobar bronchus. These differences are not related to the operation procedure because it is the same in two studies. The specific reason has been unknown up to now.

The third main finding is about the alterations of global and local measures of airflow in the tracheobronchial tree. The narrowing of the left lower lobar bronchus increases the low velocity, the wall pressure, the wall shear stress, the possibility of vortex, and the pressure drop while the inlet boundary condition is the steady constant flow rate for our present simulation. The long-term increase of these local airflow measures may result in trauma of the airway, mucosa and inflammatory response [27, 35]. With the same airflow rate, the higher pressure drop is required after lobectomy, indicating that the postoperative patients have smaller airflow rate, while the pressure drop is constant [32].

For the methodological advantages, the morphological analysis of tracheobronchial trees extracted from CT images and further CFD simulation of airflow characteristics within the trees are combined in the present work. Hence, it enables us to illustrate the relationship between alterations of airway structure and ventilation function, besides the respective ones. Via high and isotropic resolution CT images (with the voxel size of about 1 × 1 × 1 mm) and extracted tracheobronchial tree, the distortion of bronchus can be presented and bronchus kink can be diagnosed [14]. The routine postoperative follow-up CT examination does not expose patients to the additional invasive study, unlike bronchoscopy. Traditional CT and dual-energy CT applications should be expanded to image the anatomic changes and related complications for post-lobectomy patient [5, 7].

Based on the realistic and individualized tracheobronchial trees extracted from CT images, CFD provides with rich local and global information including flow velocity, wall pressure, wall shear stress, and pressure drop and flow rate ratio to the pulmonary function [14, 15]. Through strict and standard operation flow and quality control, such as the grid independence and validation, the CFD accuracy and reliability can be guaranteed. The pulmonary function test by spirometry is still the golden standard to study the changes in pulmonary function in lung cancer patients after VATS [11]. However, the concern of unnecessary risk and complex cooperation requirements for the patients limit the application of spirometry. Moreover, the changes of forced vital capacity (FVC) vary with time in the period of 3–12 months, and it reaches the maximum between 6 and 12 months [13, 36].

Regardless of the above-mentioned great advantages and findings of our study, it presents the following limitations. First, the flow rate ratio is determined according to CFD simulation without considering the CT-based lobar volume. Measuring lobar volume will help to confirm whether the ventilation and volume match well. Hyperpolarized ^3^He magnetic resonance (MR) phase-contrast velocimetry is another way of accurately measuring the airflow velocity in human airways in vivo [33]. Second, postoperative PFTs can not be collected for the concern of unnecessary risk. The scores on the cough, pain, and shortness of breath are not available, which makes it impossible to correlate our findings with these scores. The direct cause of the symptoms and guide to the patient care could not be obtained. Third, most studies on CFD simulation of airflow in human airway trees including our current study have adopted the steady flow condition for the simplification of numerical calculation and further analysis [15]. Even for the transient CFD simulation, the sine curve of the respiratory cycle is usually used as a simplified method for representing the natural respiratory cycle [26, 32]. More advanced models with the realistic boundary conditions measured by PFTs are needed. Fourth, only the patients after the left upper lobectomy are included; therefore, the comparison between different lobectomy is not achievable. It has been reported that the compensatory response after lower lobectomy is more robust than that after upper lobectomy [12], and more bronchial kinkings happen after upper lobectomy [13]. Changes in pulmonary function after right-side lobectomy are different from those after left side [11]. Finally, only the inspiratory phase CT is scanned in the current study to reduce the radiation dose and whether the inspiratory and expiratory flow will affect *θ*_R_ is still unknown. These limitations actually point out some issues for the further in-depth study.

## Conclusions

After left upper pulmonary lobectomy for lung cancer, the tracheobronchial tree will take adaptive remodeling, resulting in various structural and functional alterations. These alterations or remodelings can be favorable and unfavorable. The increase of the angle between the trachea and right main bronchus, and the resultant increase of airflow rate to the right lung are the favorable compensations of residual lung. The decrease of the angle between the trachea and left main bronchus, the sigmoidal distortion of the left main bronchus, and the stenosis of the left lower lobar bronchus are the unfavorable structural alterations. These structural alterations lead to the abnormal increase of the flow velocity, the wall pressure, the wall shear stress, the possibility of the vortex and the pressure drop, which might be associated with the realistic shortness of breath, persistent cough, and inflammation after lobectomy. Based on the morphological analysis of tracheobronchial trees extracted from CT images and further CFD simulation of airflow characteristics within the trees, all those structural and functional alterations of the tracheobronchial tree can be clarified.

## Methods

### Participants and CT images acquisition

The high-resolution CT images in DICOM format of 18 patients who underwent upper left pulmonary lobectomy for lung cancer are randomly selected out of a database of the Affiliated Zhongshan Hospital of Dalian University (Dalian, China) for a retrospective study. After anonymization, the data of each patient were given one index (LCP1–LCP18). Of the 18 patients, 12 (66.7%) were female and 6 (33.3%) were male. The mean age was 61.5 (range 50–71) years. The surgery was carried out in the period from April 2014 to October 2017. The VATS lobectomy procedure was the same as that introduced by Ueda et al. [4].

Preoperative CT images were scanned within 1 week before the lobectomy and postoperative images at 1–12 months after the lobectomy. For all acquired CT images, the tube voltage was set to 100 kV, the slice thickness was 1.0 mm, and the reconstruction matrix size was 512 × 512. The tube current, the pixel size and the number of slices were in the range of 275–673 mAs, 0.59–0.81 mm and 251–468, respectively. This study was approved by the Medical Ethics Committee of the Affiliated Zhongshan Hospital of Dalian University. Informed consent was waived because it was a retrospective review study.

### Overview of the analysis procedure

**Fig. 6:**
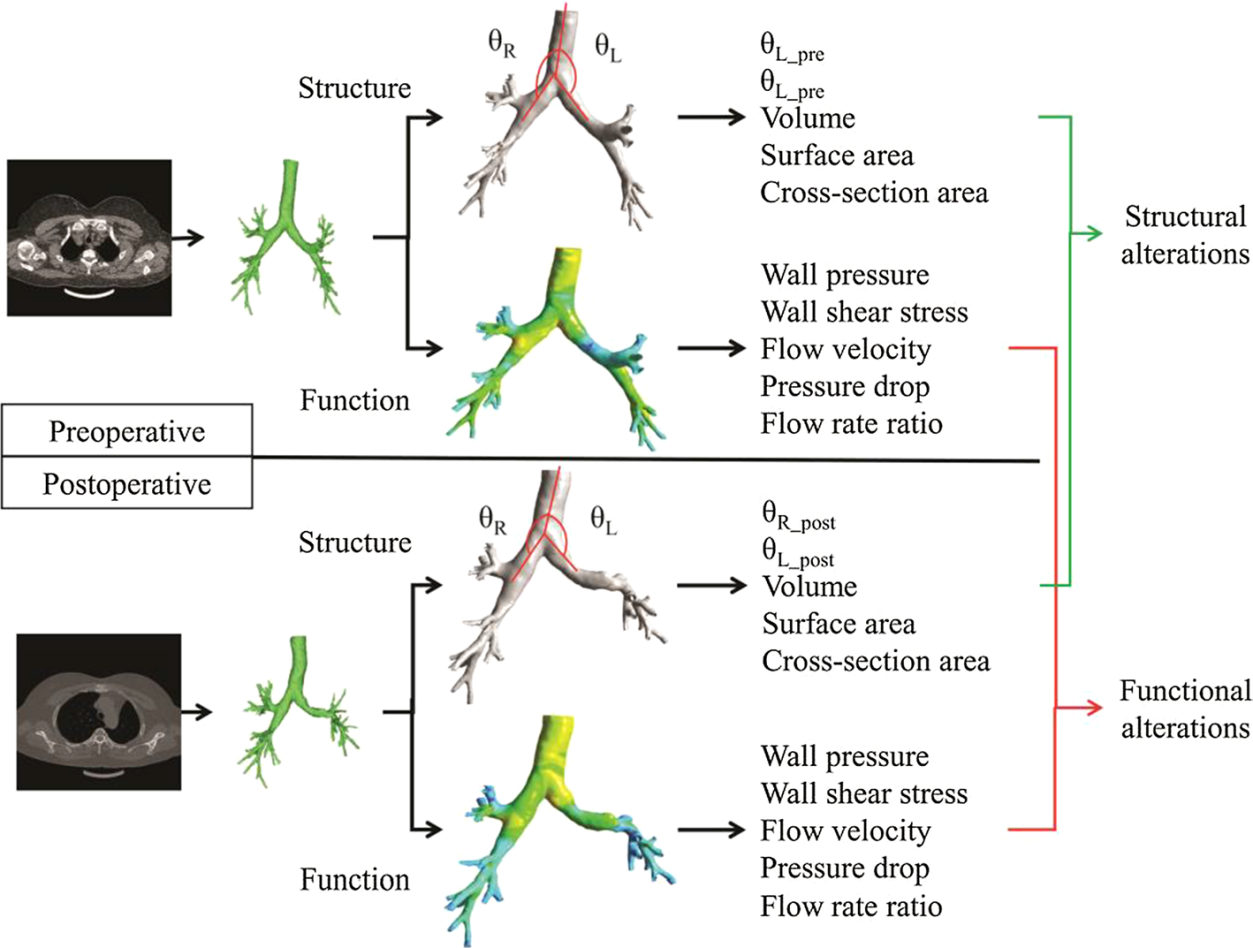
The overview of procedure in the current study

The whole analysis procedure of the present study is illustrated in Fig. 6. Using preoperative CT images, the tracheobronchial tree of each patient is extracted, and the structural measures including critical angle, surface area and volume are calculated. By CFD simulation, the measures of wall pressure, wall shear stress, flow velocity, lobar flow rate and pressure drop are obtained. After the postoperative measures are gotten similarly, the comparison between preoperative and postoperative groups produces the structural and functional alterations. The relationship between the structural and functional alterations is illustrated in coming sections.

### Structural analysis of the tracheobronchial tree

The tracheobronchial tree is extracted from the CT images using the algorithm of deep segmentation embedded in a medical imaging process software called Mimics (Materialise Corp, Belgium), and exported in the STL format. The 3D model is subsequently input into Geomagic Studio to reduce the complexity of the model. After the format of STL is converted into the X_T entity format using SolidWorks (SOLIDWORKS Corp, Waltham, USA), the tracheobronchial tree model is imported into ANSYS Workbench 15 (ANSYS Inc., Pennsylvania, USA) for CFD simulation.

As shown in Fig. 6, the angles between the trachea and the left and right main bronchus are defined as $\theta_{\text{L}}$ and $\theta_{\text{R}}$, respectively. These angles in the preoperative and postoperative models are measured and compared. The cross-sectional area growth rate is defined as

1 $$R = \left( {S_{\text{Post}} - S_{\text{Pre}} } \right)/S_{\text{Pre}} ,$$

where $S_{\text{Post}}$ is the cross-sectional area of the left lower lobar bronchus in postoperative model and $S_{\text{Pre}}$ is that in preoperative model. The volume and surface area of all the models are also measured for analysis.

### CFD analysis of the tracheobronchial tree

An advanced meshing technique is used to generate the unstructured tetrahedral elements for the highly asymmetric tracheobronchial model and path independent algorithm is used as the meshing method. The quality of the generated mesh is evaluated by the skewness and the values of skewness are found in the range of 0.8616–0.95, which eventually indicates that the mesh of the present study is acceptable. A steady breathing state with the tidal volume of 500 mL is considered as the normal adult inhalation tidal volume.

In the current study, the steady inlet velocity is set as the inlet boundary condition (BC) and the constant outlet pressure of the atmospheric pressure is set as the outlet BC [25]. As done in our previous studies [26, 27], FLUENT 16.0 is utilized to solve the governing equations of the airflow.

2 $$\frac{\partial \rho }{\partial t} + {\text{div}}\left( {\rho \upsilon } \right) = 0,$$

3 $$\rho \frac{{\partial \vec{\upsilon }}}{\partial t} = \rho \vec{F} - {\text{grad}}\vec{p} + \mu \Delta \vec{\upsilon } + \frac{\mu }{3}{\text{grad}}\left( {{\text{div}}\vec{\upsilon }} \right),$$

where $\rho$ is the fluid density, *t* is time, $\upsilon$ is the flow velocity, $\vec{\upsilon }$ is the velocity vector, $\vec{F}$ is the force vector, $\vec{p}$ is the pressure vector, $\mu$ is the viscosity of fluid. In Reynolds association numerical simulation (RANS), the above unsteady governing equations are averaged temporally.

4 $$\frac{\partial \rho }{\partial t} + \frac{\partial }{{\partial x_{j} }}\left( {\rho \bar{u}_{j} } \right) = 0,$$

5 $$\frac{\partial }{\partial t}\left( {\rho \bar{u}_{j} } \right) + \frac{\partial }{{\partial x_{j} }}\left( {\rho \bar{u}_{i} \bar{u}_{j} } \right) = - \frac{\partial P}{{\partial x_{j} }} + \frac{\partial }{{\partial x_{j} }}\left( {\mu \left( {\frac{{\partial u_{j} }}{{\partial x_{i} }} + \frac{{\partial u_{i} }}{{\partial x_{j} }}} \right)} \right) - \frac{\partial }{{\partial x_{j} }}\left( {\rho \bar{u}_{i}^{'} \bar{u}_{j}^{'} } \right) - \frac{2}{3}\frac{\partial }{{\partial x_{j} }}\left( {\mu \left( {\frac{{\partial u_{j} }}{{\partial x_{j} }}} \right)} \right) + \rho g_{i} ,$$

where $\bar{u}_{j}$ is the temporally averaged flow velocity, $\bar{u}_{i}^{'}$ and $\bar{u}_{j}^{'}$ are turbulent fluctuations, *j *= 1, 2, and 3. $x_{j}$ is the spatial coordinate and $g_{i}$ is the gravity. $\rho \bar{u}_{i} \bar{u}_{j}$ is Reynolds stress. Many turbulent models have been proposed to calculate Reynolds stress, including Eddy-Viscosity Models, Reynolds Stress Model, and Algebraic Stress Model. Here, we adopt one Eddy-Viscosity Model, i.e., the standard Low Reynolds number (LRN) *k*-$\omega$ turbulence model, where *k* and $\omega$ denote the turbulent kinetic energy and the specific dissipation rate, respectively. Meanwhile, the low-Re correction and shear flow correction are taken into account. For the inlet velocity, the turbulent intensity (*I*) is set as 5% and the turbulent viscosity ratio ($\mu_{T} /\mu$) is set as 10 [37, 38]. *I* and the turbulent viscosity $\mu_{T}$ are defined as

6 $$I = \sqrt {\bar{u}^{{{\prime }2}} + \bar{v}^{{{\prime }2}} + \bar{w}^{{{\prime }2}} } /u_{\text{avg}} ,$$

7 $$\mu_{T} = \rho C_{\mu } k^{2} /\varepsilon ,$$

where $C_{\mu } = 0.09$ and $\varepsilon$ is the rate of dissipation of turbulent energy.

The material settings and the details of the algorithm for solving the governing equations include: (1) The air is set as a Newtonian fluid with a constant density of 1.225 kg/m^3^ and a viscosity of 1.7984 × 10^−5^kg/m s. (2) A steady pressure-based solver is used. (3) The SIMPLE scheme is adopted for the pressure–velocity coupling. For the spatial discretization, the gradient is set as “Green-Gauss Cell Based”, the pressure is set as “Second Order” and the moment is set as “Second Order Upwind”. (4) The convergence criterion is set as a residual of < 10^−6^.

The relationship between the pressure drop and inlet area in straight tubes can be represented as

8 $$\Delta P = \frac{{\lambda \rho Q^{2} L}}{d}\frac{1}{{S^{2} }},$$

where $\lambda$ is the resistance coefficient along the course, $\rho$ is the density of the fluid, $Q$ is the inlet flow, $L$ is the length of the straight pipe, $d$ is the inner diameter of the round pipe and $S$ is the inlet cross-sectional area [39, 40]. Equation (8) is adopted to the tracheobronchial tree model for simplification purpose. Since there is no significant change in surface area and volume before and after lobectomy, the Eq. (8) can be simplified as

9 $$\Delta P = \frac{C}{{S^{2.5} }},$$

where $C$ is the constant. It means that the magnitude of pressure drop is inversely proportional to the inlet cross-sectional area to the power of 2.5.

For the comparison of all the above structural and functional measures, two-sample *t*-test is performed to determine whether there is a significant difference between preoperative and postoperative groups (*p* < 0.01).

### Convergence analysis

**Fig. 7:**
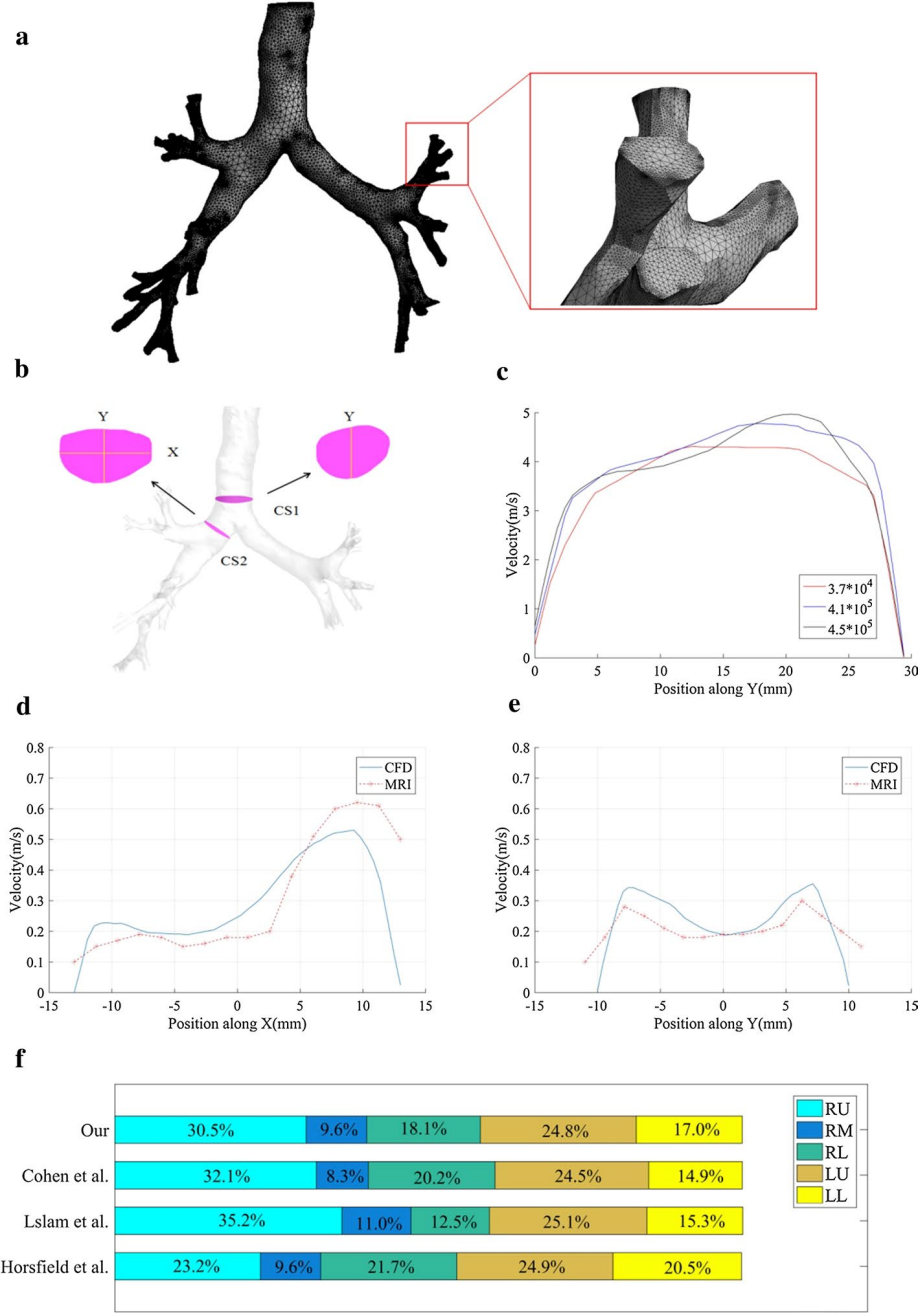
Grid independence and validation of CFD accuracy. **a** The meshes of one tracheobronchial tree model as an example. **b** Trachea cross section CS1 and bronchus cross section CS2. **c** Velocity profile along Y at CS1. **d** The velocity simulated by CFD and the results of MR gas velocity measurement at the section CS2 along X. **e** The velocity simulated by CFD and the results of MR gas velocity measurement at the section CS2 along Y. **f** The lobar distribution of airflow rate (*RU* right upper, *RM* right middle, *RL* right lower, *LU* left upper, *LL* left lower)

To study the independence of the CFD method in grid density, three different grid sizes (374,593, 412,555, and 453,954 nodes) are used to mesh all the tracheobronchial tree models. Figure 6a presents the meshes of one tracheobronchial tree model as an example where 412,555 nodes exist. The meshing quality is reasonable according to visual inspection. All other settings are the same except the grid size and we calculate and compare the airflow velocity profile along one line in the model. Specifically, two key sections (CS1 and CS2) are defined in the model (Fig. 7b). The velocity profile along Y at CS1 is calculated and compared. As shown in Fig. 7c, no significant difference in air flow velocity was observed at the three grid sizes. Comprehensively considering the calculation speed and stability, we used 412,555 nodes to mesh the model and used the same mesh density control scheme for all models.

To verify the accuracy of the CFD method, two studies were conducted and the obtained CFD simulation results were compared with the published experimental data. First, the velocity simulated by CFD at the section CS2 was compared with the results of magnetic resonance gas velocity measurement [33]. The results are shown in Fig. 7d, e, and the CFD simulation velocity along the X and Y directions of the profile are consistent with the MRI measurement flow velocity. The difference in the magnitude of the air flow velocity may be caused by the geometric differences in the model. Then, the distribution of air flow in each lobe at the same flow velocity (7.5 L/min) was studied, as shown in Fig. 7f, which is also consistent with the previous results [41–43]. The findings of the present model along with the published literature indicate that the present model is sufficiently accurate to predict the structural alterations phenomenon of the tracheobronchial model.

## Data Availability

The datasets used and/or analyzed during the current study are available from the corresponding author on reasonable request.

## Abbreviations

BC: boundary condition
CFD: computational fluid dynamics
CT: computed tomography
FEV_1_: forced expiratory volume in 1 s
FLV: functional lung volume
LCP: lung cancer patient
LRN: low Reynolds number
PFTs: pulmonary function tests
VATS: video-assisted thoracoscopic surgery
